# Combined methylmalonic acidemia and homocysteinemia presenting predominantly with late-onset diffuse lung disease: a case series of four patients

**DOI:** 10.1186/s13023-017-0610-8

**Published:** 2017-03-21

**Authors:** Jinrong Liu, Yun Peng, Nan Zhou, Xiaorong Liu, Qun Meng, Hui Xu, Shunying Zhao

**Affiliations:** 1 Department of Respiratory Medicine, Beijing Children’s Hospital, Capital Medical University, Nanlishi Road 56, Xicheng District, Beijing, People’s Republic of China; 2 Imaging Center, Beijing Children’s Hospital, Capital Medical University, Nanlishi Road 56, Xicheng District, Beijing, People’s Republic of China; 3 Department of Nephrology, Beijing Children’s Hospital, Capital Medical University, Nanlishi Road 56, Xicheng District, Beijing, People’s Republic of China

**Keywords:** Methylmalonic acidemia, Homocysteinemia, Homocystinuria, Diffuse Lung disease, Hypertension arterial pulmonary, Children

## Abstract

Combined methylmalonic acidemia (MMA) and homocysteinemia are a group of autosomal recessive disorders caused by inborn errors of cobalamin metabolism, including CblC, D, F, and J, with cblC being the most common subtype. The clinical manifestations of combined MMA and homocysteinemia vary, but typically include neurologic, developmental and hematologic abnormalities.

We report 4 children with combined MMA and homocysteinemia who presented predominantly with late-onset diffuse lung diseases (DLD). Of these, 3 accompanied by pulmonary arterial hypertension (PAH), 1 accompanied by hypertension, and 2 accompanied by renal thrombotic microangiopathy (TMA), which was confirmed by renal biopsy. This confirms combined MMA and homocysteinemia should be considered in the differential diagnosis of DLD with or without PAH or renal TMA.

## Letter to the editor

In adults, it is reported that isolated hyperhomocysteinemia may damage blood vessels, causing pulmonary arterial hypertension (PAH) and/or pulmonary thromboembolism [1, 2]. Both microangiopathy and thromboembolism can be the underlying mechanisms for pulmonary hypertension in CblC deficiency [3–8], but an association between MMA and/or homocysteinemia and diffuse lung disease (DLD) has not been broadly reported.

Here, we report a series of 4 pediatric patients with combined MMA and homocysteinemia who developed late-onset DLD. Of these, 3 presented with concomitant PAH, 1 presented with concomitant hypertension, and 2 presented with concomitant renal thrombotic microangiopathy (TMA), which was confirmed by renal biopsy. The main novelty of these cases was the predominantly pulmonary symptomatology at presentation (i.e., chronic wet cough or respiratory failure), with or without PAH (as in the first episode of “pneumonia” or “asthma” of patient 1, 3 and 4), the HRCT imaging findings, and the lack of typical neurological sequelae and ophthalmological findings. We speculate that pulmonary microangiopathy secondary to combined MMA and homocysteinemia was the primary cause of DLD in all 4 cases. Therefore, combined MMA and homocysteinemia should be considered in the differential diagnosis of DLD with or without PAH or renal TMA. This diagnosis should be investigated promptly with the proper metabolic investigations (total plasma homocysteine, plasma acylcarnitine and urine organic acid profiles), so that treatment can be initiated in a timely fashion, as lung disease secondary to this metabolic disorder will respond to appropriate treatment but may not respond to symptomatic treatment.

## Case reports

### Patient 1

A 21-month-old girl was transferred to our department with a 6-month history of slight productive cough and a 2-month history of shortness of breath. She had previously been treated with intermittent antibiotics but the cough was not resolved. Two months before admission, she had developed shortness of breath and fever, and been diagnosed with pneumonia. Echocardiography showed mild tricuspid and pulmonary valve regurgitation with a tricuspid regurgitation pressure gradient (TRPG, an estimate of pulmonary artery pressure) of 26 mmHg, which suggested that pulmonary artery (PA) pressure was normal. High-resolution computed tomography (HRCT) showed diffuse interstitial pneumonia, consolidation and pleural effusions. Fever, pulmonary consolidation and pleural effusions were resolved after treatment with antibiotics and glucocorticoids at her local hospital, but her shortness of breath and productive cough got worse.

**Fig. 1:**
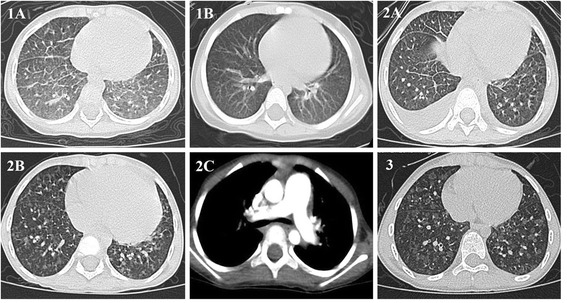
Lung CT showing the presence in both lungs of *1a* diffuse ground-glass opacification predominant in lower region of lung and areas with smooth thickening of interlobular septum (on admission; Patient 1), *1b* absence of abnormal pulmonary feature(after 1 month of treatment; Patient 1), *2a* interlobular septal thickening and bilateral pleural effusion (1 year before admission; Patient 2), *2b* diffuse poorly defined centrilobular nodules(5 days after treatment; Patient 2), *2c* pulmonary artery (PA) with an enlarged diameter exceeding the aorta(5 days after treatment; Patient 2), and *3* diffuse poorly defined ground-glass centrilobular nodules(on admission; Patient 3)

On admission to our hospital, the patient was dyspneic, with nasal alar flaring and retraction, cyanosis in the lips and nail beds. There were no rales or cardiac murmurs on auscultation of the chest. The laboratory investigations showed a white blood cell count of 14,090/mL with 66.6% neutrophils, hemoglobin (Hb) 9.8 ~ 12.6 g/dL, platelet 322 ~ 387 × 10^9^/L, serum lactate dehydrogenase (LDH) 563 IU/L and indirect bilirubin (IBIL) 33.5 μmol/L; and normal serum C-reactive protein concentration and renal function. The urinalysis showed protein in urine ranging from negative to 2+ and red blood cells (RBCs) from 0 ~ 2/HP to 2 ~ 6/HP. Arterial blood gas analysis showed type 1 respiratory failure and a mild metabolic acidosis. Sputum cultures were negative for bacterial, fungal and mycobacterial infection. An HRCT scan of her chest showed diffuse ground-glass opacification in the lower regions of the lungs, areas of smooth thickening of the interlobular septum and increased PA diameter (Fig. 1a). Echocardiography showed moderate PAH (TRPG 68 mmHg) with moderate dilation of the right atrium and ventricle, mild tricuspid and pulmonary valve regurgitation, and an ejection fraction (EF) of 74%. Based on the bilateral diffuse lung lesion of unknown reasons, mild metabolic acidosis, and her low BMI, we suspected she suffered from metabolic disease. Laboratory testing showed elevated concentration of methylmalonic acid in the serum (0.218 mg/dL) and urine (0.428 mg/dL, >420 times the reference value), and a high plasma homocysteine (Hcy) concentration (>50.0 μmol/L, reference range 5~15 μmol/L). Magnetic resonance imaging (MRI) of the brain was performed, which revealed dilated lateral ventricles with mild hydrocephalus, although she had no sigh of neurologic abnormalities. Genetic analysis confirmed a compound heterozygosity in MMACHC, with c.80A > G(p.Q27R) and c.331C > T(p.R111Ter) sequence variants, inherited from the patient’s mother and father, respectively. Combined MMA with homocysteinemia, CblC type/CblC defect/CblC deficiency was confirmed.

At diagnosis on day 7 of her admission to our hospital, treatment with captopril (3.125 mg twice daily orally) and vitamin B12 (cyanocobalamin, 0.5 mg daily intramuscularly) was started. She had improved significantly by day 10. On day 13, we added folate (5 mg once daily orally), betaine (250 mg/kg once daily orally), levocarnitine (100 mg/kg once daily orally) and vitamin B6 (10 mg/kg twice daily orally) to her therapeutic regime. Her symptoms improved gradually. One month later, HRCT of the lung showed significant improvement (Fig. 1b), but she continued to require intermittent low-flow supplemental oxygen for a further 4 months. At follow-up 1 year later, echocardiography found normal PA pressures and lung CT findings were normal. She was asymptomatic at 3 years’ follow-up.

### Patient 2

A girl aged 4 years and 7 months presented with a 1-year history of slight cough and shortness of breath. She had been observed to have lower than expected levels of physical activity, and slightly delayed intellectual and language development since birth. Echocardiography and MRI of the brain undertaken when she was 1 year old had been normal. She had been admitted to her local hospital 1 year previously due to bilateral palpebral edema, and slight cough and shortness of breath. Echocardiography undertaken at the time showed PAH, blood routine test showed a hemoglobin level of 7.7 g/dL and a platelet count of 161 × 10^9^/L, urinalysis showed proteinuria (2+) and microscopic hematuria, and HRCT showed interlobular septal thickening and bilateral pleural effusions (Fig. 1c). After treatment with bosentan, a diuretic and captopril, the palpebral edema, proteinuria and pleural effusions resolved gradually, but her cough, dyspnea and PAH did not improve, and HRCT revealed multiple nodular lesions in both lungs, so she was referred to our department.

On admission to our hospital, physical examination revealed she had cyanosis of the lips and nail beds. Chest auscultation revealed a second-degree systolic heart murmur but no rales in the lungs. Laboratory investigations detected increased serum LDH (384 IU/L) and blood urea nitrogen (BUN) concentration (11.64 mmol/L, normal range 1.7~ 7.1 mmol/L). Urinalysis revealed protein in urine 2+ and RBCs ranging from 0 ~ 2/HP to 4 ~ 6/HP. We undertook metabolic studies for her slight development delay and renal injury. Methylmalonic acid concentrations were elevated in the serum (0.294 mg/dL) and urine (0.354 mg/dL, >350 times the reference value), and a plasma Hcy concentration was >50.0 μmol/L. Therefore ophthalmologic examination was performed, which revealed bilateral macular coloboma. Echocardiography showed severe PAH (TRPG 81 mmHg) with moderate dilation of the right atrium and ventricle, EF 68%, moderate tricuspid regurgitation and mild pulmonary regurgitation. Her parents did not consent to her undergoing genetic testing. Combined MMA with homocysteinemia was clinically diagnosed.

After diagnosis on day 5 of her admission to our hospital, she was started on treatment with parenteral cyanocobalamin, levocarnitine, vitamin B6 and betaine. Her symptoms improved gradually. On day 10, contrast-enhanced lung CT showed diffuse poorly defined centrilobular nodules, and dilation of the PA to a diameter exceeding that of the aorta (Fig. 1d, e). By day 13, she was more active and was able to get out of bed. On day 15, oxygen treatment was discontinued. On day 22, echocardiography showed mild to moderate PAH (TRPG 60 mmHg) with mild dilatation of the right atrium and ventricle, mild tricuspid regurgitation and mild pulmonary regurgitation. At follow-up 2 months later at her local hospital, lung CT and echocardiography showed further substantial improvements. Unfortunately, she died suddenly 6 months later without an autopsy.

### Patient 3

A girl aged 8 years and 5 months was admitted to our hospital for investigation after a 6-year history of decreased activity and intermittent vomiting and diarrhea, and a 2-year history of renal impairment and slight productive cough. Two years before admission, she had been diagnosed with renal failure, hypertension and moderate anemia. Based on her clinical characteristics, she was suspected of having Goodpasture syndrome or vasculitis at her local hospital. At that time, urinalysis showed proteinuria 2+ and microscopic hematuria, and the lung CT revealed diffuse interstitial and parenchymal infiltration.

On admission to our hospital, physical examination revealed she had mild cyanosis of the nail beds. There were no rales or heart murmurs on auscultation of the chest. In laboratory investigations she had elevated BUN (7.73 mmol/L), serum creatinine (115 μmol/L) and IBIL (21.9 μmol/L) concentrations. Routine blood tests showed low hemoglobin (9.6 ~ 11.5 g/dL) without thrombocytopenia. The urinalysis revealed protein in urine ranging from negative to 2+ and RBCs from 0 ~ 2/HP to 2 ~ 4/HP. Diffuse poorly defined ground-glass centrilobular nodules were seen on HRCT (Fig. 1f). Echocardiography was normal, with no evidence of PAH. We undertook metabolic studies for her renal failure of unknown reasons. Methylmalonic acid concentration in the serum was 0.299 mg/dL and in the urine was 0.127 mg/dL (>120 times the reference value); plasma Hcy concentration was >50.0 μmol/L. Therefore brain MRI was performed, which revealed evident demyelinating lesions, although she had no sign of neurologic abnormalities. Genetic analyses confirmed a compound heterozygosity in *MMACHC*, with c.80A > G(p.Q27R) and c.609G > A(p.W203X) sequence variants, inherited from the patient’s mother and father, respectively. Mutation screening of atypical hemolytic uremic syndrome (HUS) associated genes (CFHR1, CFHR3, CFH, CD46, CFI, CFB, C3 and THBD), PAH associated genes (SMAD9, CAV1, KCNK3, CTEPH1, CPS1 and SARS2), pulmonary venous occlusive disease (PVOD) and pulmonary capillary hemangiomatosis (PCH) associated genes (BMPR2, EIF2AK4) showed no abnormalities. Combined MMA with homocysteinemia, CblC type was confirmed.

On day 1 after admission, she was treated with oral amlodipine besylate and fosinopril. On day 6, after diagnosis, treatment with parenteral cyanocobalamin, folate, levocarnitine, vitamin B6 and betaine was initiated. Renal biopsy undertaken on day 7 subsequently revealed mesangioproliferative glomerulonephritis and TMA with stenotic and occlusive capillaries and ischemic sclerosis. By day 10, her physical strength was improving. On day 11, bronchoscopy was performed and there was no evidence of hemosiderin-laden alveolar macrophages in the bronchoalveolar lavage fluid (BALF). She improved significantly at 9 months’ follow-up.

### Patient 4

A boy aged 7 years and 8 months was referred to our respiratory department because of a month period of mild wet cough particularly in the morning and shortness of breath. The HRCT showed mild diffuse ground-glass opacification in both lungs. Echocardiography revealed severe PAH. Asthma was suspected. After receiving a treatment with montelukast and repeated antibiotics for 1 month, and sildenafil and captopril for 15 days, his shortness of breath and PAH (mild to moderate) dramatically improved, but the cough was not resolved. When he was 4 months of age, he had hemolytic anemia without thrombocytopenia or kidney damage. Additionally, he had been diagnosed with acute glomerulonephritis with mild microscopic hematuria 1 year earlier.

On admission to our department, physical examination revealed no rales or heart murmurs on auscultation of the chest. We highly suspected he had combined MMA with homocysteinemia. Laboratory investigations showed a hemoglobin of 10.4 g/dL and mildly elevated LDH (330 IU/L), BUN (9.67 mmol/L), and Cr (71 μmol/L). Urinalysis only revealed sparse RBCs. Blood smear examination revealed sparse fragmented and deformed RBC. The serum and urinary methylmalonic acid concentrations were 0.383 mg/dL and 0.1034 mg/dL (>103 times the reference value) respectively, and plasma Hcy concentration was 193.76 μmol/L. Brain MRI revealed evident demyelinating lesions, although he had no sign of neurologic abnormalities. Genetic analyses confirmed a compound heterozygosity in *MMACHC*, with c.80A > G(p.Q27R) and c.609G > A(p.W203X) sequence variants, inherited from the patient’s father and mother, respectively. Mutation screening of atypical HUS and PAH associated genes showed no abnormalities. Combined MMA with homocysteinemia, CblC type was confirmed.

After admission, he was continuously treated with oral sildenafil and captopril. On day 4, treatment with parenteral cyanocobalamin, folate, levocarnitine, vitamin B6 and betaine was initiated. Renal biopsy undertaken on day 6 subsequently revealed TMA with stenotic capillaries, ischemic sclerosis, and membranoproliferative glomerulonephritis. Transbronchoscopic lung biopsy undertaken on day 9 subsequently revealed a thickening of the alveolar septum with a small quantity lymphatic tissue and lymphocytes. There was no evidence of hemosiderin-laden alveolar macrophages in the BALF. On day 15, his cough improved significantly and echocardiography just showed mild PAH.

In all 4 patients, metabolic investigations were suggestive of cobalamin deficiency, with elevated blood propionylcarnitine (C3) concentration as well as an elevated propionylcarnitine–acetylcarnitine (C3/C2) ratio, in the context of low or normal concentrations of methionine. Clinical characteristics and results of auxiliary examination of the 4 patients are summarized in Tables 1, 2 and 3.

**Table 1:** Demographic and clinical features, genetic evaluation and prognosis of 4 Chinese patients with combined MMA and homocysteinemia

	Patient 1	Patient 2	Patient 3	Patient 4
Gender	Female	Female	Female	Male
Age	21 months	4-years-7-months	8-years-5-months	7-years-8-months
Presentation	Cough,dyspnea	Slightly delayed intellectual and language development, cough, short of breath	Decreased activity, vomiting,diarrhea, abnormal renal function,cough	Mild wet cough and shortness of breath
Weight(kg)/PCSAG	9.0/<3rd percentile	14.0/3rd percentile	18.5/<3rd percentile	16.0/<3rd percentile
Height(cm)/PCSAG	83/30th percentile	112/90th percentile	118/<3rd percentile	123/23th percentile
Head circumference(cm)	45.1/(normal)	No record	No record	50.2/(normal)
BMI(kg/m^2^)/PCSAG	13.0/<3rd percentile	11.16/<3rd percentile	13.29/8th percentile	10.58 < 3rd percentile
Respiratory rate(breaths/min)	40	28	22	20
Heart rate(beats/min)	145	102	110	90
Blood pressure(mmHg)	90/55	90/60	130/100	95/60
Clubbed fingers	+	+	+	+
Fundus examination	Normal	Bilateral macular coloboma	Normal	Normal
MMACHC gene	c.80A > G(p.Q27R), c.331C > T(p.R111Ter)	Not available	c.80A > G(p.Q27R), c.609G > A(p.W203X)	c.80A > G(p.Q27R), c.609G > A(p.W203X)
Genotype	CblC	Not available	CblC	CblC
Follow-up	3 years	6 months	9 months	1 month
Prognosis	Asymptomatic	Improvement, but died suddenly	Improvement	Improvement

*Abbreviations*: *BMI* body mass index; *CblC* cobalamin C; *PCSAG* percentile of the children with same age and gender

**Table 2:** Laboratory features of 4 Chinese patients with combined MMA and homocysteinemia

	Patient 1	Patient 2	Patient 3	Patient 4
Hemoglobin(g/dL)	9.8 ~ 11.6	11.5 ~ 12.5	9.6 ~ 11.5	104
MCV(fL)	113.4 ~ 118.6	91.3 ~ 92.5	89.4 ~ 94.6	103.8
Platelet(×10^9^/L)	322 ~ 387	161 ~ 317	249 ~ 332	502
Urine protein	Negative ~ 2+	2+	Negative ~ 2+	Negative
Urinary erythrocytes	0-2/HP ~ 2-6/HP	0-2/HP ~ 4-6/HP	0-2/HP ~ 2-4/HP	sparse
Arterial blood gas analysis	P_a_O_2_: 27 mmHg, mild metabolic acidosis	P_a_O_2_: 49 mmHg	P_a_O_2_: 60 mmHg	P_a_O_2_: 83 mmHg
Liver function,serum albumin, cholesterol, triglyceride	Normal	Normal	Normal	Normal
IBIL (μmol/L)	33.5	10.5	21.9	8.69
BUN(mmol/L)	7.1	12.37	BUN7.73	BUN9.67
Cr (μmol/L)	38	67	115	71
eGFR (80–120 mL/min/1.73 m^2^)	81	61	37	64
Serum LDH(IU/L)	563(normal range:50 ~ 240)	384(normal range:50 ~ 240)	286(normal range:110 ~ 295)	330((normal range: 110 ~ 295)
Methionine concentrations (normal range:8.6 ~ 23.3 μmol/L)	7.6	22.6	8.4	11.1
Serous vitamin B12	Normal	Normal	Normal	Normal
Serous folate	Normal	Normal	Normal	Normal
Complement C3,C4	Normal	Normal	Normal	Normal
Thyroid hormones	Normal	Normal	Normal	Normal
Immune globulin	Normal	Normal	Normal	Normal
Lymphocyte subsets	Normal	Normal	Normal	Normal
Serum ceruloplasmin	Normal	Normal	Normal	Normal
ANA,dsDNA,ACA, ANCA	Negative	Negative	Negative	Negative
Anti-GBM Ab	Negative	Negative	Negative	Negative
Coombs test	Negative	Negative	Negative	±
Bone marrow examination	Megaloblastic anemia	Normal	Megaloblastic anemia	Normal

*Abbreviations*: *ANA* antinuclear antibody; *ACA* anticardiolipin antibody; *ANCA* antineutrophil cytoplasmic antibody; *anti*-*GBM* Ab-Anti-glomerular basement membrane antibody; *BUN* blood urea nitrogen; *Cr* creatinine; *dsDNA* double stranded DNA antibody; *eGFR* estimated glomerular filtration rate; *LDH* lactate dehydrogenase; *IBIL* indirect bilirubin concentration; *MCV* Mean Corpuscular Volume

**Table 3:** Imaging features of 4 Chinese patients with combined MMA and homocysteinemia

	Patient 1	Patient 2	Patient 3	Patient 4
PAH	+	+	—	+
EF(%)	74	68	75	77
Abdominal ultrasonography	Echo enhancement of the renal parenchyma on both sides	Mild enlargement of both kidneys	Echo enhancement and diffuse injury to the renal parenchyma on both sides	Normal
Brain MRI	Dilated lateral ventricles with mild hydrocephalus	Normal	Demyelinating lesions	Demyelinating lesions
Lung CT	Diffuse ground-glass opacification, interlobular septal thickening	Diffuse poorly defined centrilobular nodules, interlobular septal thickening	Diffuse poorly defined ground-glass centrilobular nodules	Mild diffuse ground-glass opacification,

*Abbreviations*: *EF* ejection fraction; *MRI* magnetic resonance imaging; *PAH* pulmonary arterial hypertension

## Discussion

In this case series of children with combined MMA and homocysteinemia presenting predominantly with late-onset DLD, 3 cases also had PAH and 1 had hypertension. The diagnosis was made in each of the patients mainly on the basis of clinical features of multisystem damage, elevated serum and urine methylmalonic acid concentrations, elevated plasma homocysteine concentration, and elevated blood C3 concentration and C3/C2 ratio. Combined MMA with homocysteinemia results from deficient synthesis of the coenzymes derived from vitamin B12. So far, eight variants have been described, cobalamin C (CblC) being the most prevalent. The Cb1C defect was confirmed in patients 1 (c.80A > G, c.331C > T), 3(c.80A > G, c.609G > A) and 4 (c.80A > G, c.609G > A). Patient 2 had macular coloboma, which is supportive of a cblC defect, however her parents refused to give consent for the genotyping analysis. All 3 patients carried the c.80A > G mutation which has been reported in 4 Chinese patients with TMA or PAH [9]. The c.331C > T mutation was associated with the early-onset form mainly in the French, Canadian, Acadian and Cajun populations [10, 11], and has been reported in 1 Chinese patient [12]. The c.609G > A mutation is a hot spot mutation in Chinese patients with CblC defect [12, 13] and has been reported in 2 Chinese patients with HUS [14]. Patient 3 and 4 carried compound heterozygous mutations (c.80A > G, c.609G > A), which have been reported in eight Chinese patients with early-onset of the condition [12, 13], and 1 late-onset Chinese patient with PAH and renal TMA [9]. However, to date, the most frequent MMACHC mutations associated with PAH and renal TMA were c.271dupA, c.276G > T, and c.565C > A in Western countries [4–7, 15, 16].

Combined MMA and homocysteinemia is a multisystemic disorder that can lead to damage to the central nervous system, retina, liver, kidneys and bone marrow (http://www.ncbi.nlm.nih.gov/books/NBK1328/). In our series, all patients had evidence of renal injury, hematologic and neurologic abnormalities to some extent;patient 2 also had bilateral macular coloboma, which are also common in combined MMA and homocysteinemia. Cardiomyopathy, both dilated, hypertrophic and non compaction, as well as microangiopathy have been described in patients affected with this group of conditions [17]. There have been 2 reports of pulmonary embolism and PAH in children with cobalamin C defect [3, 18], 7 of PAH in patients(including 1 adult) with cobalamin C defect [4–7, 9, 15, 16], 1 of PAH in newborn with MMA [19]. PVOD has been described as histological diagnosis in 3 patients with CblC defect [6, 16]. Contrast-enhanced lung CT was performed in patient 2 and didn’t show thin pulmonary venous or pulmonary artery embolism. DLD improved significantly after treatment in all patients, which didn’t suggest PVOD. In addition the lung abnormalities identified on imaging were not sufficiently severe to have caused PAH. Therefore we consider PAH was caused by combined MMA and homocysteinemia.

In this study, we aimed to show that there may be a relationship between combined MMA and homocysteinemia and DLD in children, although interstitial lung disease has been reported in association with other syndromes such as Niemann-Pick disease, as well as pulmonary alveolar proteinosis. No other causes of DLD, such as connective tissue disease, alveolar hemorrhage syndromes, pulmonary vasculitis, hypersensitivity pneumonitis, drug induced interstitial pneumonia, or infection were detected in any of the patients. Thus we think that in these cases, it is highly possible that DLD was caused by combined MMA and homocysteinemia, and not by PAH, based on the following reasons. Respiratory symptoms arose before or at the time of PAH diagnosis in the first 2 patients and in patient 4. Additionally, DLD existed in the absence of PAH in patient 3, despite lung imaging being similar to that in patient 2. Furthermore, DLD improved substantially in all patients when they were treated for MMA and homocysteinemia, but patient 2 did not improve when she was treated with bosentan.

*Post mortem* examination of 4 patients with combined MMA and homocysteinemia found severe vascular lesions, with renal damage characteristic of TMA [20, 21], thromboemboli in the pulmonary circulation [18, 21], and massive endothelial proliferation in pulmonary post-capillary venules [16]. In the renal biopsy undertaken in the last 2 patients we observed TMA with stenotic and occlusive capillaries. Further, there have been reports showing this association [6, 7, 15, 16]. Additionally, the first 2 patients also had renal injury (including proteinuria and microscopic hematuria) and anemia. Furthermore patient 1 had an elevated level of serum LDH and IBIL, and patient 2 had an elevated level of serum LDH, which suggested that both of them might have atypical renal TMA especially patient 1. Renal TMA may be underestimated especially in China because its features are too subtle. None of the 4 patients presented with thrombocytopenia, which was consistent with the report by Komhoff M [6]. There was also imaging evidence of diffuse pulmonary microangiopathic lesions in all 4 of our patients. Therefore, we also speculate that the damage of vascular endothelial cell function (including pulmonary vessels) was triggered by intracellular and systemic changes by MMACHC variants. This was particularly marked in the presence of increased serum homocysteine concentration and could increase the permeability of pulmonary capillaries. The resulting vasoconstriction, vascular smooth muscle cell proliferation, and microthrombus formation, especially pulmonary TMA [1, 7], caused the presence of diffuse ground-glass opacification and centrilobular nodules in the lungs.

In this paper, DLD was diagnosed by lung imaging. DLD is consist of many kinds of lung diseases, including pulmonary vasculopathy.

Our study has several limitations. We did not undertake blood smear examination in the first 3 patients, because of our poor awareness of TMA. Additionally, we did not undertake lung biopsy in the first 3 patients, as it was not warranted by the severity of patients 1 and 2, and the parents of patient 3 declined to give consent. Patient 4 underwent transbronchoscopic lung biopsy, and the histologic findings only showed a thickening of the alveolar septum without vascular structure, owing to the small tissue sample. These patients were not treated with hydroxocobalamin, which was unavailable in mainland China. We used TRPG to estimate PA pressures rather than undertake right heart catheterization, which is substantially more invasive.

## Conclusions

CblC defect has biochemical (combined MMA and homocysteinemia), radiological (DLD) and cardiological (PAH) characteristics. Our study suggested combined MMA and homocysteinemia be considered a potential reversible cause of DLD and PAH. Prompt recognition, diagnosis and treatment of CblC defect may reverse not only lung damage, but may prevent other complications associated with CblC defect (renal microangiopathy), and that it should be suspected not only in children, but in adults with similar findings.

## Abbreviations

BALF: Bronchoalveolar lavage fluid
BMI: Body mass index
BUN: Blood urea nitrogen
cblC: Cobalamin C
DLD: Diffuse lung diseases
EF: Ejection fraction
Hcy: Homocysteine
HRCT: High-resolution computed tomography
HUS: Hemolytic uremic syndrome
IBIL: Indirect bilirubin
LDH: Lactate dehydrogenase
MMA: Methylmalonic Acidemia
MRI: Magnetic resonance imaging
PA: Pulmonary arterial
PAH: Pulmonary arterial hypertension
PCH: Pulmonary capillary hemangiomatosis
PVOD: Pulmonary venous occlusive disease
RBCs: Red blood cells
TMA: Thrombotic microangiopathy
TRPG: Tricuspid regurgitation pressure gradient
